# Sexually transmitted infections among pregnant Syrian refugee women seeking antenatal care in Lebanon

**DOI:** 10.1093/jtm/taae058

**Published:** 2024-04-09

**Authors:** Sasha A Fahme, Iman Fakih, Ali Ghassani, Mostafa El-Nakib, Laith J Abu-Raddad, Jeffrey D Klausner, Ghina R Mumtaz

**Affiliations:** Department of Epidemiology and Population Health, American University of Beirut, P.O. Box 11-0236, Beirut, Lebanon; Department of Epidemiology and Population Health, American University of Beirut, P.O. Box 11-0236, Beirut, Lebanon; Community Research Department, Amel Association, Beirut, Lebanon; National AIDS Program, Beirut, Lebanon; Infectious Disease Epidemiology Group, Weill Cornell Medicine-Qatar, Cornell University, Qatar Foundation – Education City, P.O. Box 24144, Doha, Qatar; World Health Organization Collaborating Centre for Disease Epidemiology Analytics on HIV/AIDS, Sexually Transmitted Infections, and Viral Hepatitis, Weill Cornell Medicine–Qatar, Cornell University, Qatar Foundation–Education City, P.O. Box 24144, Doha, Qatar; Department of Population Health Sciences, Weill Cornell Medicine, Cornell University, New York, NY 10065, USA; Department of Public Health, College of Health Sciences, QU Health, Qatar University, P.O. Box 2713 Doha, Qatar; College of Health and Life Sciences, Hamad bin Khalifa University, P.O. Box 34110, Doha, Qatar; Keck School of Medicine, University of Southern California, Los Angeles, CA 90033, USA; Department of Epidemiology and Population Health, American University of Beirut, P.O. Box 11-0236, Beirut, Lebanon; Center for Infectious Diseases Research, Faculty of Medicine, American University of Beirut, P.O. Box 11-0236, Beirut, Lebanon

**Keywords:** Chlamydia, gonorrhoeae, trichomoniasis, forced displacement, pregnancy, epidemiology

## Abstract

The prevalence of *Chlamydia trachomatis*, *Neisseria gonorrhoeae* and *Trichomonas vaginalis* was determined among 431 pregnant Syrian refugee women seeking antenatal care in Lebanon. Low prevalence at 0.5% for chlamydia, 0.2% for trichomoniasis and 0.0% for gonorrhoeae was detected, suggesting a low burden of sexually transmitted infection in this population.

Untreated sexually transmitted infections (STIs) may have devastating effects on maternal and neonatal health. While routine antenatal STI screening is adopted in many high-income countries, there remain significant gaps in detection and treatment throughout the Global South, including in the Middle East and North Africa (MENA). Widespread conflict in this region has resulted in the protracted displacement of millions of women who may consequently experience poor sexual and reproductive health.^1^^,^^2^

In Lebanon, a country with the world’s highest per capita refugee population, antenatal care engagement among Syrian refugee women is estimated to be >80%,^3^ making antenatal care clinics a pragmatic setting in which to screen this otherwise difficult-to-access population for curable STIs. Curable STIs may represent a modifiable risk factor to reduce maternal and neonatal morbidity among pregnant refugee women, but the epidemiology of STIs is unknown in this vulnerable population. We aimed to estimate the prevalence of and risk factors for *Chlamydia trachomatis*, *Neisseria gonorrhoeae* and *Trichomonas vaginalis* among pregnant Syrian refugee women engaged in antenatal care in Lebanon.

This cross-sectional study adhered to a standardized protocol.^4^^,^^5^ Ethical approval was obtained from the Institutional Review Board of the American University of Beirut (ID: BIO-2020-0163). Participants were recruited from five public antenatal care clinics in refugee-dense areas within the Greater Beirut (*N* = 2), Beqaa (*N* = 2) and Tyre (*N* = 1) regions of Lebanon, which provide subsidized antenatal care to low-income populations. The clinics in Greater Beirut and Tyre are situated in refugee camp-adjacent urban slums, and those in the Beqaa are located in semi-rural villages in close proximity to the Lebanese–Syrian border.

All adult pregnant Syrian refugee women at <34 weeks of gestation seeking antenatal care in the participating clinics were eligible to participate. Additional eligibility criteria included: willingness to be tested for *C. trachomatis*, *N. gonorrhoeae* and *T. vaginalis*; willingness to receive treatment if diagnosed; willingness to attend a test-of-cure if treated and ability to provide written informed consent. A convenience sample was pursued due to the absence of a reliable and representative sampling frame. Participants were consecutively enrolled from February to September 2022 in Beirut and Tyre and from July to August 2023 in the Beqaa. Of 437 eligible women screened, 431 (98.6%) consented to participation.

After obtaining written informed consent, trained interviewers administered an Arabic-language survey collecting sociodemographic information. Participants provided a 50 cc urine specimen in a sterile container for testing. Urine specimens were tested for *C. trachomatis* and *N. gonorrhoeae* using the GeneXpert® CT/NG assay, and for *T. vaginalis* using the GeneXpert® TV assay (Cepheid, Sunnyvale, California, USA). These nucleic acid amplification tests methods have U.S. Food and Drug Administration clearance for use on female urine specimens and demonstrate 97.6% sensitivity and >99.4% specificity for *C. trachomatis* detection, 95.6% sensitivity and >99.8% specificity for *N. gonorrhoeae* detection, 99.5–100.0% sensitivity and 99.4–99.7% specificity for *T. vaginalis* detection. Controls were run on the Cepheid GeneXpert® equipment for each assay. Automated reports were reviewed to establish precision and accuracy of the findings. Women diagnosed with any of the three infections and their partners were offered standard treatment.

Demographic data were analysed descriptively and stratified by setting using chi-squared test for categorical variables and *t* test for continuous variables. Bivariate log-binomial regression was used to explore sociodemographic associations across urban and rural settings. STI prevalence data were reported as frequencies and proportions across the overall sample. Analyses were conducted using R 4.3.0.

There were 431 participants aged 18 to 44 years (mean = 25.6 ± SD = 5.8) from urban (*n* = 318, 73.8%) and rural (*n* = 113, 26.2%) settings enrolled in this study. Table 1 outlines the sociodemographic characteristics of participants. The overwhelming majority (99.5%) were married and 61.2% were married prior to reaching 18 years of age. The mean number of lifetime sex partners was 1.1 (SD = 0.3), with 8.4% of women reporting a history of >1 sex partner. On average, participants residing in the Beqaa had spent more time in forced displacement in Lebanon (*P* < 0.001) and reported greater household crowding (*P* < 0.05). When compared to their urban counterparts, women recruited in the Beqaa had 4.9 times greater risk of residing in a refugee camp (95% CI 3.4 to 7.1); 2.8 times greater risk of having never been enrolled in school (95% CI 1.7 to 4.6); and 4.1 times greater risk of being in a polygamous marriage (95% CI 1.8 to 9.6).

**Table 1:** Participants’ sociodemographic characteristics, Syrian pregnant refugee women in Lebanon

	All participants	Urban	Rural	*P* value
	*N* = 431	*N* = 318	*N* = 113	
	*n* (%)	*n* (%)	*n* (%)	
Age (years)				
18–24	213 (49.4)	154 (48.4)	59 (52.2)	0.651
25–29	113 (26.2)	85 (26.7)	28 (24.8)	
30–34	62 (14.4)	49 (15.4)	13 (11.5)	
>34	43 (10.0)	30 (9.4)	13 (11.5)	
Mean ± SD [median]	25.6 ± 5.8 [25.0]	25.6 ± 5.8 [25.0]	25.6 ± 5.9 [24.0]	0.949
Residence				
Non-camp	338 (78.4)	284 (89.3)	54 (47.8)	<0.001
Camp	93 (21.6)	34 (10.7)	59 (52.2)	
Years in Lebanon				
<5	133 (30.9)	111 (34.9)	22 (19.5)	0.012
5–10	247 (57.3)	171 (53.8)	76 (67.3)	
>10	51 (11.8)	36 (11.3)	15 (13.3)	
Mean ± SD [median]	6.7 ± 4.1 [7.0]	6.3 ± 4.1 [6.0]	7.8 ± 3.8 [9.0]	<0.001
Education level				
None	54 (12.5)	27 (8.5)	27 (23.9)	<0.001
8^th^ grade or lower	303 (70.3)	231 (72.6)	72 (63.7)	
Secondary school or university	74 (17.2)	60 (18.9)	14 (12.4)	
Marital status				
Married	429 (99.5)	316 (99.4)	113 (100.0)	0.613
Separated	2 (0.5)	2 (0.6)	0 (0.0)	
Number of times married				
1	395 (91.6)	294 (92.5)	101 (89.4)	0.323
>1	36 (8.4)	24 (7.5)	12 (10.6)	
Mean ± SD [median]	1.1 ± 0.3 [1.0]	1.1 ± 0.3 [1.0]	1.1 ± 0.3 [1.0]	
Age at first marriage (years)				
<15	47 (10.9)	35 (11.0)	12 (10.6)	0.680
15–18	217 (50.3)	165 (51.9)	52 (46.0)	
19–24	131 (30.4)	92 (28.9)	39 (34.5)	
>24	36 (8.4)	26 (8.2)	10 (8.8)	
Mean ± SD [median]	18.3 ± 3.6 [18.0]	18.3 ± 3.5 [17.0]	18.5 ± 3.6 [18.0]	0.550
Polygamous marriage				
Yes	22 (5.1)	9 (2.8)	13 (11.5)	<0.001
No	407 (94.9)	307 (97.2)	100 (88.5)	
Household crowding index^*^				
1–2	136 (31.6)	108 (34.0)	28 (24.8)	0.082
>2	295 (68.4)	210 (66.0)	85 (75.2)	
Mean ± SD [median]	3.2 ± 1.7 [3.0]	3.1 ± 1.6 [3.0]	3.6 ± 1.9 [3.0]	0.034

Abbreviation: SD: standard deviation.

^*^Number of persons in the household divided by number of rooms in the house.

We detected two cases of chlamydia, corresponding to an estimated prevalence of 0.5% (95% CI 0.1% to 1.7%) and one case of trichomoniasis, corresponding to an estimated prevalence of 0.2% (95% CI 0.0% to 1.4%). All three participants were recruited from clinics in Beirut. We did not identify any cases of gonorrhoeae or of co-infections.

This is the first study of STI epidemiology among pregnant Syrian refugee women, who comprise the largest population of forcibly displaced women globally. The study population was characterized by low education, early marriage and prolonged displacement within crowded settings in Lebanon. Our findings suggest, among pregnant Syrian refugee women in Lebanon, a prevalence of curable STIs lower than what is observed among women in other MENA countries,^6^ but comparable to what is observed in Lebanon.^7^ Data on the prevalence of curable STIs among other various groups of travellers, including migrants, are scarce. However, available evidence suggests a generally low prevalence, albeit slightly higher than the findings of this study.^8^^,^^9^

Notably, our study population consists of pregnant women engaged in monogamous sexual partnerships in the context of marriage. The MENA region has been long characterized by conservative social norms that discourage premarital sexual relationships. Indeed, only few women reported >1 lifetime sexual partner and only in the context of previous marriages. Our results are aligned with findings from a study in Pakistan, a similarly conservative MENA country.^4^

While rural-dwelling participants were observed to have greater vulnerability in terms of displacement-specific factors, it is not clear in this small study whether such factors confer a greater STI risk. The inverse correlation between conflict and STIs has been previously described in Sub-Saharan Africa, in part due to reduced mobility and smaller sexual networks.^10^ While Syrian refugee women in Lebanon similarly experience constrained mobility,^1^ the impact on sexual networks and STI transmission dynamics among urbanized refugees warrants further examination.

This study has several weaknesses. Firstly, we were unable to conduct risk factor analyses due to the low STI prevalence. Secondly, this study adhered to a standardized protocol that was limited to screening for chlamydia, gonorrhoeae and trichomoniasis.^5^ One limitation of this protocol is the exclusion of *Treponema pallidum* testing, which has implications for congenital syphilis. Also, syphilis testing would have necessitated the collection of another biological sample (blood), which may have discouraged participation. We similarly did not test for HIV, due to pervasive HIV stigma, which also may have deterred participation, as well as reliable evidence that suggests low HIV prevalence in the general population.^2^ Future studies are needed to understand the burden of syphilis, HIV and other STIs among this refugee population.

Nevertheless, the study also has a number of strengths. We developed rigorous standard operating protocols utilizing nucleic acid amplification testing with quality control measures on all laboratory analyses. While the lack of census data in Lebanon presents considerable challenges to recruiting a probability-based sample, given high rates of antenatal care retention, our geographically diverse sample may accurately reflect the larger population of pregnant Syrian refugee women engaged in care in Lebanon.

In conclusion, pregnant Syrian refugee women in Lebanon are a low sexual-risk population that likely experience a low burden of curable STIs. While universal STI screening may not be indicated, prospective data among larger samples are needed to identify predictors of STIs among Syrian refugee women in Lebanon to potentially support targeted STI screening efforts.

## Sources of funding

This work was supported by UK Research and Innovation as part of the Global Challenges Research Fund [grant number ES/P010873/1]. G.R.M., I.F. and L.J.A. acknowledge support by the Biomedical Research Program at Weill Cornell Medicine in Qatar.

## Data Availability

The data underlying this article will be shared on reasonable request to the corresponding author.

## Notes

Preliminary findings were presented at the 23 International Union against Sexually Transmitted Infections (IUSTI) World Congress, Victoria Falls, Zimbabwe. September 5, 2022.

## References

[1] Samari G . Syrian refugee Women's health in Lebanon, Turkey, and Jordan and recommendations for improved practice. World Med Health Policy 2017; 9:255–74. 29051840 10.1002/wmh3.231 PMC5642924

[2] Mumtaz GR , Chemaitelly H, AlMukdad S et al. Status of the HIV epidemic in key populations in the Middle East and North Africa: knowns and unknowns. The Lancet HIV 2022; 9:e506–16. 35777412 10.1016/S2352-3018(22)00093-5

[3] Benage M , Greenough PG, Vinck P, Omeira N, Pham P. An assessment of antenatal care among Syrian refugees in Lebanon. Confl Health 2015; 9:8. 25741381 10.1186/s13031-015-0035-8 PMC4349304

[4] Chaudry AE , Chaudhri R, Kayani A et al. Acceptability and feasibility of screening pregnant women for sexually transmitted infections in Rawalpindi, Pakistan. Int J STD AIDS 2021; 32:940–5. 34009081 10.1177/09564624211007681

[5] Shannon CL , Bristow C, Hoff N et al. Acceptability and feasibility of rapid chlamydial, gonococcal, and Trichomonal screening and treatment in pregnant women in 6 Low- to middle-income countries. Sex Transm Dis 2018; 45:673–6. 29528996 10.1097/OLQ.0000000000000832 PMC6129444

[6] Smolak A , Chemaitelly H, Hermez JG, Low N, Abu-Raddad LJ. Epidemiology of chlamydia trachomatis in the Middle East and North Africa: a systematic review, meta-analysis, and meta-regression. Lancet Glob Health 2019; 7:e1197–225. 31402004 10.1016/S2214-109X(19)30279-7

[7] Hanna J , Yassine R, El-Bikai R et al. Molecular epidemiology and socio-demographic risk factors of sexually transmitted infections among women in Lebanon. BMC Infect Dis 2020; 20:375. 32460721 10.1186/s12879-020-05066-8 PMC7251815

[8] Adachi M , Takemura S. Outcomes of systemic screening for syphilis, gonorrhoea and chlamydia trachomatis among immigrant visa applicants migrating from Japan to the US in 2016-2019. J Travel Med 2022; 29. 10.1093/jtm/taac084 35880852

[9] Shiferaw W , Martin BM, Dean JA et al. A systematic review and meta-analysis of sexually transmitted infections and blood-borne viruses in travellers. J Travel Med 2024. 10.1093/jtm/taae038 PMC11149723 38438164

[10] Shannon K , Kaida A, Rachlis B, Lloyd-Smith E, Gray G, Strathdee SA. Reconsidering the impact of conflict on HIV infection among women in the era of antiretroviral treatment scale-up in sub-Saharan Africa: a gender lens. AIDS 2008; 22:1705–7. 18753938 10.1097/QAD.0b013e328308de0e

